# A48 PATIENT OUTCOMES OF A COMPLEX POLYP ASSESSEMNT COMMITTEE IMPLEMENTATION

**DOI:** 10.1093/jcag/gwae059.048

**Published:** 2025-02-10

**Authors:** P Gallagher, H Petropolis, M Stewart, S Patel, D Farina, A Kohansal

**Affiliations:** Department of Medicine, Dalhousie University, Halifax, NS, Canada; Department of Medicine, Dalhousie University, Halifax, NS, Canada; Department of Medicine, Dalhousie University, Halifax, NS, Canada; Department of Medicine, Dalhousie University, Halifax, NS, Canada; Department of Medicine, Dalhousie University, Halifax, NS, Canada; Department of Medicine, Dalhousie University, Halifax, NS, Canada

## Abstract

**Background:**

Over the past two decades, the endoscopic approach to managing complex colonic polyps has shifted from surgery to advanced endoscopic techniques, including endoscopic mucosal resection (EMR) and Endoscopic Submucosal Dissection. Endoscopic methods are less invasive than surgery, reducing morbidity, mortality, and cost, but require additional time, training, and expertise. Despite guidelines recommending EMR as the first line option, surgical resection for complex non-invasive polyps remains common in Canada. A 2019 retrospective study revealed that 12.5% of large non-malignant polyps in Nova Scotia were treated with surgical resection. Subsequently, a Complex Polyp Assessment Committee (CxPAC) was established to assess polyps suitable for endoscopic resection, aiming to reduce unnecessary surgical procedures.

**Aims:**

The project aims to examine the early outcomes of programmatic assessment and management of complex colorectal polyps though CxPAC.

**Methods:**

A retrospective chart review was completed on all patients referred to Nova Scotia’s CxPAC between January 2022 and February 2024. Descriptive statics were calculated.

**Results:**

In a 24-month study period, 99 patients were referred to the CxPAC, with 88 suitable for complex polypectomy. Among the 10 patients not deemed appropriate for the procedure, 7 were directed to surgery prior to any endoscopic intervention. The referral indication was polyp size (65.7%), size with challenging position (16.2%), challenging position alone (6.1%), residual/recurrent polyp (7.1%), and scarring/poor lift (5.1%). The mean patient age was 66 (SD, 12), and the mean polyp size was 37mm (SD, 17). There were 18 patients referred for surgical management: 2 based on referral image review, 10 after endoscopic or post-resection pathology appearance positive for invasive carcinoma, 1 after perforation, and 5 after incomplete resection. Follow-up endoscopies have been performed on 61 patients. Among the 40 patients deemed clear during follow-up endoscopy, 8 did not have a biopsy at the polypectomy site. Of the 32 patients who did undergo biopsies, 20 exhibited normal mucosa, 8 showed reactive changes/scarring post-polypectomy, 2 were diagnosed with tubular adenomas, 1 with a sessile serrated adenoma, and 1 with a hyperplastic polyp. There were 9 instances of severe adverse events related to complex polypectomy: 5 cases of post-polypectomy bleeding, 2 perforations, and 2 emergency department visits due to abdominal pain.

**Conclusions:**

The CxPAC has been effective for assessing and managing complex non-malignant colonic polyps, with 88.6% of patients able to avoid surgery. While endoscopic resection of complex polyps is a safer alternative to surgery, procedural risks remain, highlighting the importance of additional training on the part of the endoscopists and a detailed discussion and consent process with the patients.

**Funding Agencies:**

None

